# CAD-RADS may underestimate coronary plaque progression as detected by serial CT angiography^[Author-notes jeab215-FM2]^

**DOI:** 10.1093/ehjci/jeab215

**Published:** 2021-10-23

**Authors:** Bálint Szilveszter, Borbála Vattay, Melinda Bossoussou, Milán Vecsey-Nagy, Judit Simon, Béla Merkely, Pál Maurovich-Horvat, Márton Kolossváry

**Affiliations:** MTA-SE Cardiovascular Imaging Research Group, Heart and Vascular Center, Semmelweis University, 68 Városmajor st, 1122 Budapest, Hungary; MTA-SE Cardiovascular Imaging Research Group, Heart and Vascular Center, Semmelweis University, 68 Városmajor st, 1122 Budapest, Hungary; MTA-SE Cardiovascular Imaging Research Group, Heart and Vascular Center, Semmelweis University, 68 Városmajor st, 1122 Budapest, Hungary; MTA-SE Cardiovascular Imaging Research Group, Heart and Vascular Center, Semmelweis University, 68 Városmajor st, 1122 Budapest, Hungary; MTA-SE Cardiovascular Imaging Research Group, Heart and Vascular Center, Semmelweis University, 68 Városmajor st, 1122 Budapest, Hungary; MTA-SE Cardiovascular Imaging Research Group, Heart and Vascular Center, Semmelweis University, 68 Városmajor st, 1122 Budapest, Hungary; MTA-SE Cardiovascular Imaging Research Group, Heart and Vascular Center, Semmelweis University, 68 Városmajor st, 1122 Budapest, Hungary; Medical Imaging Centre, Semmelweis University, 2 Korányi Sándor st, 1083 Budapest, Hungary; MTA-SE Cardiovascular Imaging Research Group, Heart and Vascular Center, Semmelweis University, 68 Városmajor st, 1122 Budapest, Hungary

**Keywords:** coronary artery disease, CT angiography, coronary plaque progression

## Abstract

**Aims:**

We wished to assess whether different clinical definitions of coronary artery disease (CAD) [segment stenosis and involvement score (SSS, SIS), Coronary Artery Disease—Reporting and Data System (CAD-RADS)] affect which patients are considered to progress and which risk factors affect progression.

**Methods and results:**

We enrolled 115 subsequent patients (60.1 ± 9.6 years, 27% female) who underwent serial coronary computed tomography angiography (CTA) imaging with >1year between the two examinations. CAD was described using SSS, SIS, and CAD-RADS. Linear mixed models were used to investigate the effects of risk factors on the overall amount of CAD and the effect on annual progression rate of different definitions. Coronary plaque burdens were SSS 4.63 ± 4.06 vs. 5.67 ± 5.10, *P* < 0.001; SIS 3.43 ± 2.53 vs. 3.89 ± 2.65, *P* < 0.001; CAD-RADS 0:8.7% vs. 0.0% 1:44.3% vs. 40.9%, 2:34.8% vs. 40.9%, 3:7.0% vs. 9.6% 4:3.5% vs. 6.1% 5:1.7% vs. 2.6%, *P* < 0.001, at baseline and follow-up, respectively. Overall, 53.0%, 29.6%, and 28.7% of patients progressed over time based on SSS, SIS, and CAD-RADS, respectively. Of the patients who progressed based on SSS, only 54% showed changes in CAD-RADS. Smoking and diabetes increased the annual progression rate of SSS by 0.37/year and 0.38/year, respectively (both *P* < 0.05). Furthermore, each year increase in age raised SSS by 0.12 [confidence interval (CI) 0.05–0.20, *P* = 0.001] and SIS 0.10 (CI 0.06–0.15, *P* < 0.001), while female sex was associated with 2.86 lower SSS (CI −4.52 to −1.20, *P* < 0.001) and 1.68 SIS values (CI −2.65 to −0.77, *P* = 0.001).

**Conclusion:**

CAD-RADS could not capture the progression of CAD in almost half of patients with serial CTA. Differences in CAD definitions may lead to significant differences in patients who are considered to progress, and which risk factors are considered to influence progression.

## Graphical Abstract

**Graphical Abstract:**
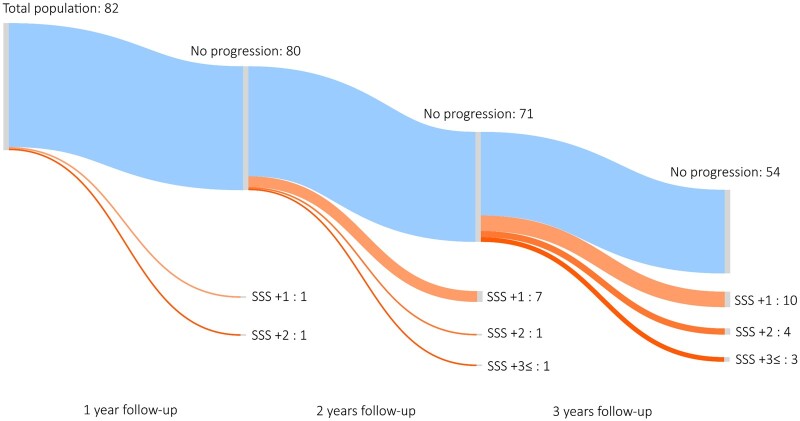

## Introduction

Coronary artery disease (CAD) is a chronic disease with dynamic temporal changes in plaque size and composition.^1,2^ Coronary computed tomography angiography (CTA) is a robust, non-invasive diagnostic test to characterize CAD.^3,4^ Coronary CTA allows the accurate assessment of the presence, extent and severity of coronary atherosclerosis, and has received class I recommendation (level of evidence B) for the evaluation of CAD among patients with stable chest pain in the latest guidelines.^5^ Recent studies have proposed CAD progression as a prognosticator of adverse events, irrespectively from stenosis severity using CTA.^1^ However, many clinical definitions and metrics of coronary plaque progression (PP) have been proposed.^6,7^ Nevertheless, there are still limited data on the predictors of coronary PP with respect to different metrics of CAD quantity.

Therefore, we aimed to evaluate the predictors of CAD progression using different clinical definitions of plaque burden on serial computed tomography (CT) imaging. In addition, we sought to compare the identification of PP by three different semiquantitative definitions of CAD including the stenosis classification of the Coronary Artery Disease—Reporting and Data System (CAD-RADS).

## Methods

### Patient population

**Figure 1:**
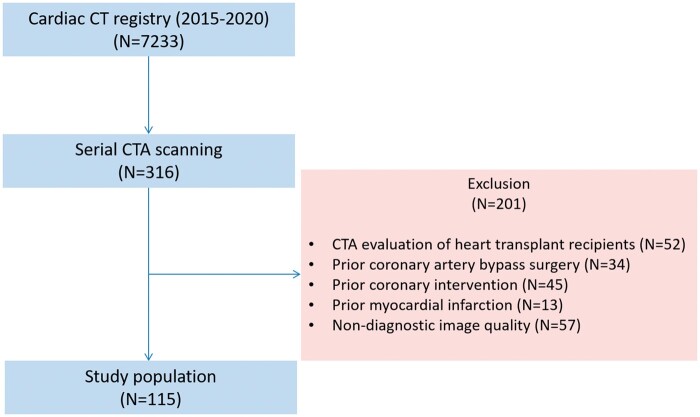
Flow chart of the study. CT, computed tomography; CTA, computed tomography angiography.

From 7233 patients in our structured reporting registry of coronary CTA, 316 had at least two coronary CTAs with at least 1 year between the two examinations using the same 256-slice scanner (Philips Brilliance iCT, Best, The Netherlands) between 1 January 2015 and 6 January 2020. Indications for serial testing were the planning of radiofrequency ablation in patients with paroxysmal atrial fibrillation (23.4%) or recurrent angina after initial CTA (76.6%). Exclusion criteria for our analysis were myocardial infarction, percutaneous coronary intervention, heart transplantation, or coronary bypass graft surgery prior to any CTA imaging and non-diagnostic CT image quality. Overall, we found 115 symptomatic and stable patients who met the inclusion and exclusion criteria (*Figure 1*). Anamnestic, anthropometric data and CTA findings were recorded in a structured reporting platform (Axis, Neumann Medical Ltd. Budapest, Hungary).

The study was approved by the institutional review board and informed consent was waived due to the retrospective nature of the study. All procedures used in this study were in accordance with local and federal regulations, and the Declaration of Helsinki.

### Demographics and comorbidities

Medical chart review was performed to gather data on patient demographics and comorbidities. Both at baseline and follow-up, patients underwent detailed interview on risk factors, medical history, and medication. Hyperlipidaemia was defined as having elevated plasma cholesterol levels (total cholesterol >200 mg/dL) or the use of lipid-lowering therapy. Hypertension was defined as systolic blood pressure >140 mmHg and/or diastolic blood pressure >90 mmHg or antihypertensive medication use verified by medical records. Smoking was defined as prior tobacco use within 1 year prior CTA (both baseline and follow-up time point evaluated). Diagnosis of diabetes mellitus (DM) was established based on elevated plasma glucose levels (fasting plasma glucose ≥ 126 mg/dL; HbA1c ≥ 6.5%) or the use of antidiabetic medication or insulin therapy. Statin use was recorded at baseline and follow-up at the time of the CT scanning and was defined as the use of statin at the time of the scans.

### CT Image acquisition

We performed prospectively triggered CTA of the heart according to the guidelines of the SCCT.^8^ Per os metoprolol was administered in case of heart rate exceeded 65 beats per minute 1 h before the coronary CTA examination and if not contraindicated. All patients received 0.8 mg of sublingual nitroglycerine prior to the CTA and intravenous beta-blocker was also administered if heart rate was still above 60 beats/min. Image acquisition was performed at diastole (75–81% of the R–R interval) or at systole (37–43% of the R–R interval) if heart rate was above 70 bpm despite premedication. The following scanner settings were used: 270 ms rotation time, 128 × 0.625 mm collimation, tube voltage of 100–120 kVp, and tube current of 200–300 mAs based on patient’s body mass index (BMI). Axial images were reconstructed with 0.4 mm slice thickness using iterative reconstruction.

### Coronary CTA analysis

A single reader with 7 years of experience in cardiovascular CT imaging (B.S.) assessed the location, morphology, and severity of coronary lesions using an 18-segment coronary tree model.

Coronary plaque was defined on the CTA based on former publications by Mahabadi *et al.* and Achenbach *et al.*^9,10^ Calcified and non-calcified plaques were defined as any discernible structure with a density of ≥130 and <130 Hounsfield units, respectively, which were assigned to the vessel wall in at least two independent image planes. Coronary segments with a diameter of >1.5 mm were analysed. The reader evaluated baseline and follow-up images simultaneously to detect changes in plaque composition (on visual evaluation) or stenosis severity and was blinded to patient characteristics, CTA date, comorbidities, and medical treatment.

To quantify total CAD burden segment involvement score (SIS) (sum of all coronary segments affected with plaque) and segment stenosis score (SSS) (sum of coronary segments involved with plaque weighted with stenosis severity: 0%: 0, 1–24%: 1, 25–49%: 2, 50–69%: 3, 70–99%:4, 100%: 5) were calculated in all 18 segments of all patients at baseline and follow-up scans.^6^ Inter-reader reproducibility of SSS and SIS was assessed previously.^11^ CAD-RADS stenosis categories (0: 0%, 1: 1–24%, 2: 25–49%, 3: 50–69%, 4A 70–99%, 4B: Left main >50% or three-vessel disease, 5: 100%) were assessed according to the CAD-RADS consensus document.^12^ The presence, extent, and severity for all lesions were entered into a structured reporting platform that automatically generated CAD-RADS clinical recommendation (Axis, Neumann Medical Ltd. Budapest, Hungary)^12^ based on these conditional inputs of the reader.

### Statistical analysis

Continuous variables are presented as mean and standard deviation, whereas categorical parameters are presented as frequency with percentages. Paired sample *t*-test was used to compare parameters describing coronary plaque burden of the two CTA examinations. First, we used linear regression analysis to identify predictors of annualized PP. Furthermore, we applied a more complex approach using linear mixed models to analyse repeated observations at non-standardized intervals.^13–15^

By analysing intra-individual changes over time we can simultaneously estimate the effect of a predictor on (i) overall amount of the outcome (SSS, SIS, and CAD-RADS) and (ii) effect on annual progression rate.^15,16^ Using this information, the model is able to provide an estimate on the effect of a covariate on overall amount of the outcome, irrespective of time (e.g. statin users tend to have more plaque, as statin is initiated in patients with increased plaque burdens), but it also provides estimates on how the covariate affects temporal changes (e.g. statin users will have slower progression as compared to the average). This method also already accounts for the total plaque burden at baseline, therefore no additional adjustment is needed in the models. We calculated univariate linear mixed models to assess the effect of each predictor on the outcome (CAD definitions) and annual progression of the outcome. If either effect had a *P*-value <0.10, we included that predictor in a multivariate model. Clinical predictors of coronary PP were included in the models as predictors with SIS, SSS, and CAD-RADS as outcomes. Inter-observer reproducibility was assessed in 25 patients by two observers using weighted kappa. We selected five plaques per stenosis category (minimal 1–24%, mild 25–49%, moderate 50–69%, severe 70–99%, and occluded 100%), including one plaque per patient. The *k* values were interpreted as follows: 0.00–0.20 poor; 0.21–0.40 fair; 0.41–0.60 moderate; 0.61–0.80 good; and 0.81–1.00 excellent agreement.

All analyses were conducted in the R environment v3.6.1 and STATA v13.0. A two-sided *P*-value smaller than 0.05 was considered statistically significant.

## Results

The baseline characteristics of the 115 enrolled patients (mean age 60.1 ± 9.6 years, 27% female) are summarized in *Table 1*. On average, 2.6 ± 1.1 years has passed between the CTA examinations. A total of 1763 coronary artery segments were evaluated at both time points. Mean effective radiation dose was 5.07 and 5.09 mSv (*P* = 0.822) for baseline and follow-up.

**Table 1:** Patient characteristics

Patient data	Study population (*N* = 115)
Age (years)	60.1 ± 9.6
BMI (kg/m^2^)	28.4 ± 4.2
Female gender, *n* (%)	27 (23.5)
Cardiovascular risk factors at baseline, *n* (%)	
Hypertension	87 (75.7)
Diabetes mellitus	15 (13.0)
Dyslipidaemia	63 (54.8)
Smoking	13 (11.3)
Family history of premature CAD	29 (25.2)
Statin use	47 (35.7)

BMI, body mass index; CAD, coronary artery disease.

### Characteristics of coronary PP

A total of 105 (91.3%) patients had any CAD at baseline. The remaining 10 patients (8.7%) had no plaque on baseline and all developed minimal stenosis on follow-up (CAD-RADS 1). We detected no progression of disease in 54 patients with CAD (46.7%), furthermore, plaque regression was not observed in this patient population.

We detected a total of 397 plaques at baseline vs. 449 plaques at follow-up in the total population. Regarding plaque types, we found 142 vs. 154 calcified, 175 vs. 203 partially calcified, and 80 vs. 92 non-calcified plaques on baseline vs. follow-up scans, respectively. Comparing the first and second coronary CTA images we found that SSS, SIS, and CAD-RADS significantly increased on the follow-up images: SSS 4.63 ± 4.06 vs. 5.67 ± 5.10, *P* < 0.001; SIS 3.43 ± 2.53 vs. 3.89 ± 2.65, *P* < 0.001; CAD-RADS 0: 8.7% vs. 0.0% 1: 44.3% vs. 40.9%, 2: 34.8% vs. 40.9%, 3: 7.0% vs. 9.6% 4: 3.5% vs. 6.1% 5: 1.7% vs. 2.6%, *P* < 0.001, at baseline and follow-up, respectively (see *Table 2*). The average annual progression rate was 0.41 ± 0.62 for SSS and 0.18 ± 0.34 for SIS. Any progression in SSS, SIS, and CAD-RADS was found in 53.0%, 29.6%, and 28.7% of all cases. Importantly, among patients without progression in CAD-RADS during the follow-up period, 34.1% and 17.1% had progression in SSS and SIS, respectively. Of the patients who progressed based on SSS, only 54% showed changes in CAD-RADS scores.

**Table 2:** Coronary plaque burden at baseline and follow-up

	1. Scan (*n* = 115)	2. Scan (*n* = 115)	*P*-value
SSS	4.63 ± 4.06	5.67 ± 5.10	<0.001
SIS	3.43 ± 2.53	3.89 ± 2.65	<0.001
CAD-RADS severity, *n* (%)			<0.001
0	10 (8.7)	0 (0.0)	
1	51 (44.3)	47 (40.9)	
2	40 (34.8)	47 (40.9)	
3	8 (7.0)	11 (9.6)	
4	4 (3.5)	7 (6.1)	
5	2 (1.7)	3 (2.6)	

CAD-RADS, Coronary Artery Disease—Reporting and Data System; SIS, segment involvement score; SSS, segment stenosis score.

**Figure 2:**
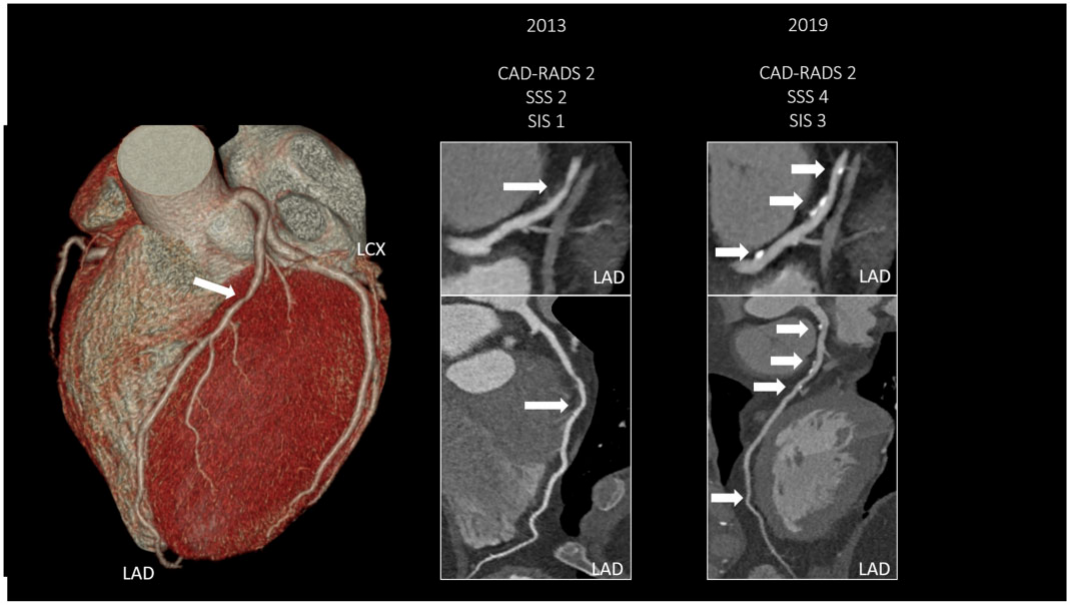
Representative case of plaque progression. A 51-year-old male patient underwent serial CTA scanning due to stable chest pain. In 2013, the patient had one affected coronary segment (mid-LAD) with plaque. The figure shows the LAD with a non-calcified plaque leading to mild stenosis (CAD-RADS 2, SSS 2, SIS 1). We detected coronary plaque progression on the proximal and distal LAD segment of LAD 6 years later, including *de novo* calcified plaque development. The total segment involvement and segment stenosis scores were both higher on follow-up (SSS 4 and SIS 3), however, based on the worst stenosis CAD-RADS score of 2 was assigned. CAD-RADS, Coronary Artery Disease—Reporting and Data System; LAD, left anterior descending artery; LCX, Left circumflex artery; SIS, segment involvement score; SSS, segment stenosis score.

**Figure 3:**
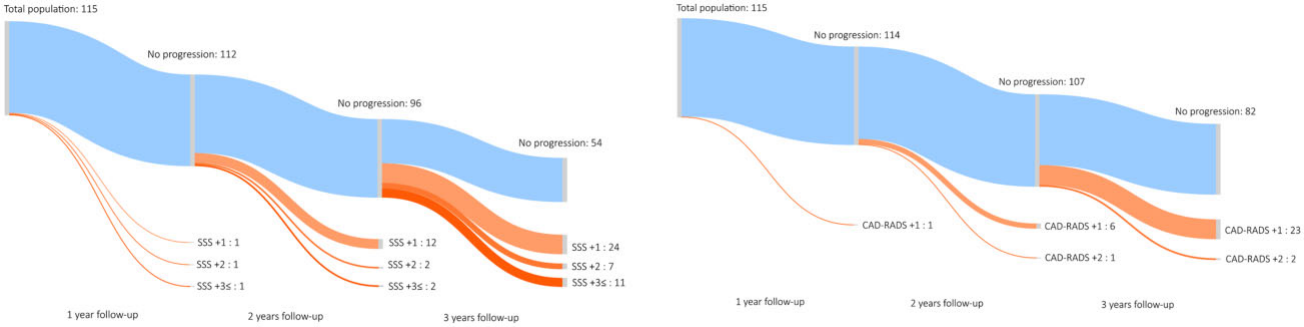
Sankey diagram depicting coronary plaque progression based on SSS and CAD-RADS. Patient-based progression of coronary atherosclerosis using SSS and CAD-RADS severity is depicted on the Sankey diagram. This type of flow diagram depicts the number of patients with progression during follow-up, where the width of the arrow is proportional to the flow rate (number of patients). No progression was found in 54 patients’ SSS and in 82 patients’ CAD-RADS during the follow-up period (marked with light blue). The number of patients with increase in SSS and CAD-RADS are depicted at 1, 2, and ≥3 years based on serial CTA imaging (orange). CAD-RADS substantially underestimates changes in disease severity and extent of CAD. During the follow-up period we could identify three patients with SSS increase at 1 year (rapid progression of CAD), whereas most patients progressed later at 3 or more years. CAD-RADS, Coronary Artery Disease—Reporting and Data System; SSS, segment stenosis score.

**Figure 4:**
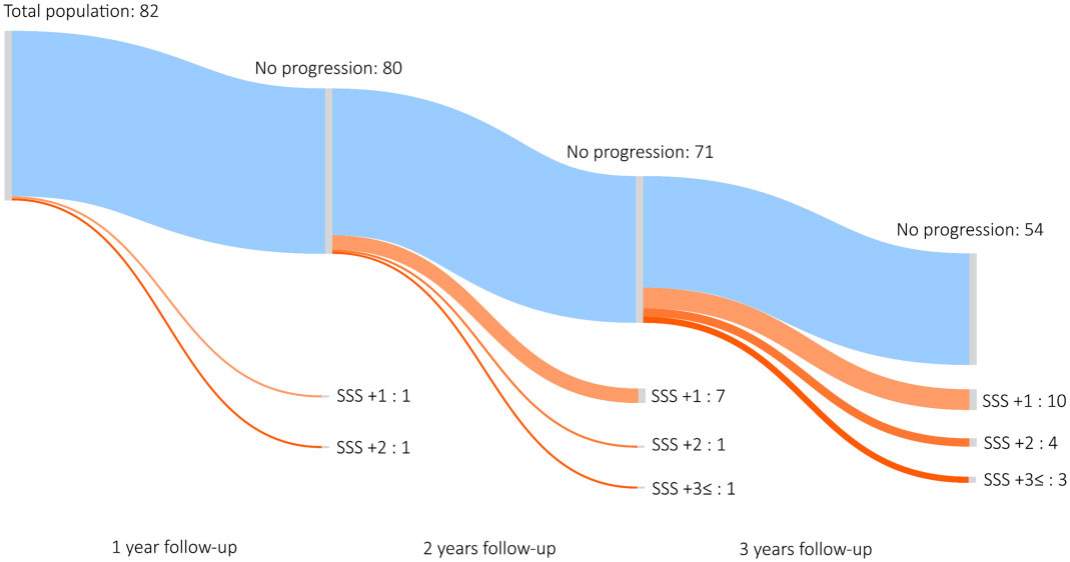
Sankey diagram depicting coronary plaque progression based on SSS among those with no progression in CAD-RADS classification for lesion severity. A total of 82 patients in our study did not progress based on CAD-RADS severity, although a large proportion of patients demonstrated coronary plaque progression based on SSS. Increasing number of patients developed higher stenosis scores during follow-up period: 2 patients had higher SSS at 1 year, 9 patients had higher SSS at 2 years, and 17 patients had higher SSS at 3 years or more. Four patients had substantial increase in SSS (three or more) despite no changes in CAD-RADS classification. SSS, segment stenosis score.

Representative case of CAD progression is seen on *Figure 2*, changes in SSS and CAD-RADS during follow-up are summarized on a Sankey diagram (*Figure 3*). SSS-based progression is depicted among those with no changes in CAD-RADS severity on a Sankey diagram (*Figure 4*).

We found excellent agreement across stenosis categories evaluating 25 plaques (5 from each stenosis category) by two observers (weighted kappa = 0.903).

### Predictors of coronary PP

Linear regression models were carried out to identify predictors of PP described as annualized progression of SSS or SIS. Age was associated with PP based on SSS [β = 0.013, confidence interval (CI) 0.01–0.024, *P* = 0.034] but showed no association with SIS progression (*P* = 0.784). Importantly, none of the other predictors showed any association with the annualized progression rate of SSS, SIS, or CAD-RADS (all *P* > 0.05). However, this approach does not account for the temporal changes of covariates over time and does not account for the one’s baseline value, therefore we also analysed our data using linear mixed models.

### Linear mixed models for the analysis of coronary PP and progression rate

Univariate linear mixed models showed that age and gender affect the value of SSS and SIS in our population, whereas diabetes increased the annual progression rate of SSS by 0.34/year (CI 0.01–0.66; *P* = 0.036) (see *Table 3*).

**Table 3:** Univariate analysis of predictors of plaque progression using linear mixed models

Predictors	SSS						SIS						CAD-RADS severity					
	Univariate model						Univariate model						Univariate model					
	Effect on SSS			Effect on annual progression			Effect on SIS			Effect on annual progression			Effect on CAD-RADS			Effect on annual progression		
	β	95% CI	*P*-value	β	95% CI	*P*-value	β	95% CI	*P*-value	β	95% CI	*P*-value	β	95% CI	*P*-value	β	95% CI	*P*-value
Age	**0.11**	**0.04–0.19**	**0.004**	**0.01**	**0.00–0.02**	**0.035**	**0.09**	**0.05–0.14**	**<0.001**	0.00	0.00–0.01	0.701	0.01	0.00–0.03	0.140	0.00	−0.01 to 0.00	0.510
Gender	**−2.31**	**−4.04 to −0.58**	**0.010**	−0.08	−0.34 to 0.17	0.516	**−1.38**	**−2.44 to −0.31**	**0.013**	−0.01	−0.15 to 0.13	0.898	−0.34	−0.76 to 0.07	0.110	0.02	−0.07 to 0.10	0.713
BMI	−0.02	−0.13 to 0.08	0.651	−0.01	−0.03 to 0.02	0.609	0.04	−0.02 to 0.10	0.182	−0.01	−0.02 to 0.01	0.254	0.00	−0.03 to 0.04	0.901	0.00	−0.01 to 0.01	0.730
Hypertension	0.15	−1.22 to 1.51	0.834	0.19	−0.06 to 0.44	0.136	0.28	−0.51 to 1.07	0.479	0.02	−0.11 to 0.16	0.740	−0.12	−0.48 to 0.25	0.523	0.05	−0.03 to 0.13	0.229
Diabetes mellitus	1.39	−0.84 to 3.61	0.225	**0.34**	**0.02–0.66**	**0.036**	0.81	−0.56 to 2.18	0.251	0.06	−0.11 to 0.24	0.473	0.17	−0.36 to 0.70	0.527	0.05	−0.06 to 0.15	0.377
Dyslipidaemia	0.64	−0.47 to 1.75	0.264	−0.12	−0.34 to 0.09	0.269	0.28	−0.37 to 0.93	0.390	−0.05	−0.17 to 0.07	0.427	0.13	−0.18 to 0.44	0.412	−0.06	−0.14 to 0.01	0.083
Smoking	0.67	−0.55 to 1.88	0.281	0.33	0.00–0.65	0.050	0.64	−0.08 to 1.37	0.083	0.09	−0.08 to 0.26	0.286	0.06	−0.38 to 0.49	0.794	0.05	−0.05 to 0.15	0.311
Statin use	−0.11	−0.92 to 0.70	0.785	0.15	−0.08 to 0.38	0.203	−0.16	−0.61 to 0.30	0.498	0.05	−0.07 to 0.18	0.413	0.12	−0.15 to 0.38	0.378	−0.05	−0.12 to 0.03	0.241

Univariate linear mixed models demonstrating the effects of risk factors on SSS, SIS, and CAD-RADS. Significant predictors are marked in bold letters. The effect of each risk factor on the total amount of SSS, SIS, and CAD-RADS is described by the model. Yearly progression rate of each predictor is also described by the effect of progression rate. All models incorporate the baseline value of CAD metrics.

BMI, body mass index; CAD-RADS, Coronary Artery Disease—Reporting and Data System; CI, confidence interval; SIS, segment involvement score; SSS, segment stenosis score.

On multivariate analysis, patients who smoked had significantly increased annual progression rate of SSS by 0.37/year (CI 0.07–0.67, *P* = 0.017) and higher total extent of CAD as described by SIS as compared with non-smokers (β = 0.77, CI 0.06–1.50, *P* = 0.034). DM increased the annual progression rate of SSS by 0.38/year (CI 0.07–0.69, *P* = 0.016). Age and gender affected the total amount of SSS and SIS (*P* ≤ 0.001, all): 1-year increase in age lead to an estimated increase of 0.12 (CI 0.05–0.20, *P* = 0.001) in SSS and 0.10 (CI 0.06–0.15, *P* < 0.001) in SIS. Female gender is associated with an average of 2.86 lower SSS (CI −4.52 to −1.20, *P* < 0.001) and 1.68 lower SIS (CI −2.65 to −0.71, *P* = 0.001) than male gender. Importantly, CAD-RADS was not influenced by any cardiovascular risk factor (all *P* ≥ 0.05). Detailed results of multivariate analysis are summarized in *Table 4*.

**Table 4:** Multivariate analysis of predictors of plaque progression using linear mixed models

Predictors	SSS				SIS				CAD-RADS Severity			
	Effect on SSS		Effect on annual progression		Effect on SIS		Effect on annual progression		Effect on CAD-RADS		Effect on annual progression	
	β (95% CI)	*P*-value	β (95% CI)	*P*-value	β (95% CI)	*P*-value	β (95% CI)	*P*-value	β (95% CI)	*P*-value	β (95% CI)	*P*-value
Age	**0.12 (0.05–0.20)**	**0.001**	**0.01 (0.01–0.03)**	**0.013**	**0.10 (0.06–0.15)**	**<0.001**	0.00 (0.00–0.01)	0.369		NS		NS
Female gender	**−2.86 (−4.52 to −1.20)**	**<0.001**	−0.23 (−0.47 to 0.01)	0.06	**−1.68 (−2.65 to −0.71)**	**0.001**	−0.03 (−0.17 to 0.11)	0.646		NS		NS
Smoking	1.16 (−0.03 to 2.35)	0.06	**0.37 (0.07–0.67)**	**0.017**	**0.77 (0.06–1.50)**	**0.034**	0.10 (−0.07 to 0.27)	0.263		NS		NS
Diabetes mellitus	1.27 (−0.86 to 3.40)	0.24	**0.38 (0.07–0.69)**	**0.016**								

Multivariate linear mixed models including independent predictors of coronary plaque progression. Significant predictors are marked in bold letters.

CAD-RADS, Coronary Artery Disease—Reporting and Data System; CI, confidence interval; NS, Non-significant; SIS, segment involvement score; SSS, segment stenosis score.

## Discussion

In our longitudinal observation cohort study, we found that age and gender influenced the severity and the extent of CAD after correcting for the effects of other conventional cardiovascular risk factors. Smoking and DM were significant factors increasing annual progression rates of SSS, reflecting both the severity and the extent of the disease. Importantly, among those with increased SSS and SIS on follow-up CTA, in 46% and 41% of patients the CAD-RADS severity classification did not change. On the other hand, none of the risk factors seem to influence CAD-RADS, the currently recommended clinical classification framework for reporting CAD.

Coronary atherosclerosis is a dynamic and progressive disease that may lead to the obstruction of the coronary lumen and induce ischaemia.^17^ CTA is a uniquely suited imaging modality to monitor changes in the extent and severity of CAD and underlying plaque composition.^18^ Quantifying coronary plaque burden improves risk assessment using both semi-quantitative (SSS, SIS) or quantitative (volumetric) plaque metrics.^19,20^ Recent studies incorporated the degree of stenosis, plaque morphology, and SSS for the detection of PP. Although quantitative plaque analysis (volumetric change in atheroma burden) is not used routinely in clinical setting, this biomarker has been increasingly utilized for monitoring anti-atherosclerotic drug therapy.^21^

Despite increasing number of studies utilizing CT imaging for the detection of the plaque development, there are limited data on the predictors of atherosclerosis progression. Moreover, there is a huge variety of definitions in use to quantify CAD progression and to characterize coronary atherosclerosis on CTA per se. Motoyama *et al.*^1^ defined disease progression as either an increase in stenosis by at least 1 grade or an increase in the remodelling index ratio of >1.1, and found that increase in plaque burden might be the strongest predictor of adverse events. In other studies, progression was defined based on newly diagnosed cases with 50% or more coronary stenosis by the person-years of follow-up,^22^ whereas Gu *et al.*^23^ used coronary calcium score, SSS, and SIS for describing progression. More recent investigations focused on quantitative plaque analysis and defined PP based on volumetric changes (≥10%) compared to baseline volume^24^ or simply evaluated the change in plaque volume.^25^ Notably, most of these changes in plaque volume are relatively small and would not change clinical scoring systems such as SSS or CAD-RADS classification and clinical decision-making. Other limitations of using volumetric plaque analysis include insufficient image quality, lack of standardized protocols regarding acquisition protocols and iterative reconstruction.^26,27^ Furthermore, many concerns have been raised regarding the inter-vendor, scan–rescan, inter-software, and inter-reader variability of quantitative plaque analysis which limits its widespread clinical use.^28–30^

Importantly, the methodology of the aforementioned studies differed as compared to our analysis, since we are the first to use linear mixed models to define the effect of risk factors on CAD metrics and the progression rate. Most of the prior studies analysed two timepoints defining progression as the change in mean values of plaque parameters of a given population, but this approach might not account for the one’s baseline value (i.e. individuals with more plaque may progress faster unless normalizing for the baseline plaque volume) or the possible changes in covariates over time (i.e. someone may develop DM over the follow-up period). Moreover, some studies enrolled patients with non-standardized follow-up times for serial scanning, which—also based on our observation of PP rate—can significantly influence results. Linear mixed models provide flexible modelling of intra-subject changes and enable to assess overall and individual patterns in time. This method enables to assess the impact of predictors in both timepoints on the outcomes.^15^

To incorporate the main findings of coronary CTA in a unified and standardized framework, experts of the field have proposed the use of the CAD-RADS classification in clinical practice. Maroules *et al.*^31^ found high inter-observer agreement for both experienced and early career readers when assessing CAD-RADS categories, except for vulnerability. Importantly, the currently used CAD-RADS classification scheme only describes the plaque with the largest degree of stenosis and does not account for the extent of disease. For example, patients with mild or moderate stenosis (CAD-RADS category 2 or 3)—who are classified as non-obstructive patients—could also demonstrate extensive CAD affecting more than four coronary segments. Bittencourt *et al.*^32^ demonstrated that extensive, but non-obstructive CAD patients have comparable risk profile as those with obstructive CAD. CAD burden scores (SSS and SIS) used in current study also incorporate the number of segments affected with plaques. *Figure 2* shows a representative case of our study highlighting the importance of using different scores when describing PP. In this case, the patient had *de novo* plaques on two additional segments whereas the CAD-RADS category remained the same and thus might create the false impression that CAD did not progress throughout the years. Coronary PP is a strong prognosticator of events and therefore should also be implemented in CAD-RADS recommendation. Proper risk assessment is crucial to better define a subset of patients who require a more aggressive secondary prevention therapy or downstream testing.

We detected substantial changes in SSS and SIS during follow-up imaging; however, the CAD-RADS classification was not able to identify changes in the extent and severity of CAD and thus might not recognize increased risk for future events. CAD-RADS classification has been proposed to guide further testing and secondary prevention therapy; however, based on our data, it would miss almost every second patient with increased plaque severity or extent which have been previously shown to increase adverse events. The use of CAD-RADS underestimates coronary PP as it heavily focuses on the worst plaque of a given patient and only includes three-vessel obstructive ≥70% disease in grade 4B when assessing plaque burden. Coronary CTA provides, however, rich information on the distribution, extent, and vulnerability of coronary atherosclerosis. The novel version of the classification scheme could improve risk assessment by combining the stenosis severity and plaque burden, moreover, experts of the field also suggested adding flow-limiting lesions and coronary artery calcium score information as well. Further outcome studies are warranted to evaluate the prognostic value of the current CAD-RADS classification and possible future versions in terms of plaque severity vs. the extent of CAD. Also, PP should be included in the classification and its predictive value should be tested in large contemporary cohort of outpatients with stable angina.

Prior investigations report conflicting results regarding the predictors of PP, which might originate from the differences in the definition of progression and the use of CAD scoring systems. Increased PP might be promoted by male gender, obesity, high low-density lipoprotein cholesterol level, DM, and smoking.^33,34^ Among conventional cardiovascular risk factors, DM, BMI, and smoking induced PP in other studies.^35,36^ Smoking can promote atherogenesis involving pathways of inflammation, endothelial dysfunction, platelet function, cholesterol metabolism, and thrombotic factors.^37–39^ We also found that male gender was associated with larger SSS and SIS, moreover, patients who smoked had larger extent of CAD. Among cardiovascular risk factors smoking and DM were related to the progression rate of SSS using linear mixed models.

We acknowledge the limitations of our study. First, we retrospectively analysed patients who underwent serial CTA at a single tertiary centre for Cardiology with different time interval between the two coronary CTA examinations. We therefore used linear mixed models that account for differences in follow-up times and provide detailed information on the factors of PP. Another limitation is the incidental statin use without routine cholesterol measurements and strict control of adherence to the drug. Also, prospective, multicentre trials are warranted to analyse whether the observed discrepancies between different CAD definitions could influence patient outcomes. Furthermore, smoking was defined as prior tobacco use within 1 year prior CTA reflecting current smoking and not life-long accumulated hazards of smoking.

## Conclusion

CAD-RADS did not capture the progression of CAD in almost half of stable angina patients with serial CT imaging. Age and gender influenced segment stenosis and plaque extent as described by segment stenosis and involvement scores. Smoking and diabetes affected the progression rate of PP based on SSS. Differences in CAD definitions may lead to significant differences in patients who are considered to progress. Therefore, unified plaque metrics are needed that are capable to properly describe the extent and severity of CAD for CT-based risk prediction and clinical management.

## Funding

This study was supported by the National Research, Development and Innovation Office of Hungary (NKFIA; NVKP_16-1-2016-0017 National Heart Program). The research was financed by the Thematic Excellence Programme (Tématerületi Kiválósági Program, 2020-4.1.1.-TKP2020) of the Ministry for Innovation and Technology in Hungary, within the framework of the Therapeutic Development and Bioimaging programmes of the Semmelweis University.

## Data Availability

The data underlying this article will be shared on reasonable request to the corresponding author.
